# Vaccination Administration Training Across Pharmacy Degree Levels: A Global Survey of Pharmacy Educators

**DOI:** 10.3390/pharmacy14070144

**Published:** 2026-09-30

**Authors:** Kimberly McKeirnan, Ilse Truter, Teri-Lynne Fogarty

**Affiliations:** 1 Department of Pharmacy, Nelson Mandela University, Gqeberha 6031, South Africa; ilse.truter@mandela.ac.za (I.T.); teri-lynne.fogarty@mandela.ac.za (T.-L.F.); 2 Department of Pharmacotherapy, Washington State University, Spokane, WA 99202, USA

**Keywords:** pharmacy education, vaccination training, paediatric vaccination, pharmacy curriculum, immunisation education, global survey

## Abstract

**Background/Objectives:** Pharmacist vaccination authority has expanded globally over the past two decades, but education and training for vaccine administration have not consistently kept pace. Evidence on curricular content and delivery has emerged from individual countries and institutions in isolation, with no global, cross-regional comparison across pharmacy degree levels. This study aimed to describe the content, delivery format, assessment, and certification practices for vaccination administration training across Bachelor’s, Master’s, and Doctoral pharmacy programmes globally, and to assess pharmacy educators’ perceptions of the adequacy of current training. **Methods:** A cross-sectional survey of pharmacy educators (*n* = 81) from 35 countries was administered via Qualtrics between January and April 2026. Responses were analysed descriptively (frequencies and percentages). Master’s and Doctoral responses were compared with Bachelor’s using McNemar’s exact test, with McNemar’s Odds Ratio as the effect-size statistic. **Results:** Fewer than a third of institutions offering training included paediatric administration technique at any degree level (28.6% Bachelor’s, 14.3% Master’s, 20.0% Doctoral), and educators rated their institution’s education as significantly less adequate for paediatric vaccination than adult vaccination (Wilcoxon signed-rank *W* = 5.5, *n* = 71, *p* < 0.001, *r* = 0.52). Training status did not differ significantly between Bachelor’s (41.9% taught) and postgraduate (32.4% taught) levels (McNemar χ^2^(1, *n* = 74) = 1.33, *p* = 0.248, OR = 0.59). Roughly half of students at every degree level completed training without receiving a formal certificate, and financial cost and limited curriculum time were the most frequently cited barriers to training (41.4% and 37.1% of institutions, respectively). **Conclusions:** Accreditation bodies and pharmacy programmes should prioritize structured certification pathways so that more students can receive formal certification and training.

## 1. Introduction

Pharmacists are recognised globally as an important part of the immunisation workforce, and vaccination authority has expanded across many countries over the past two decades [1,2]. A recent literature review has identified that education and training for vaccine administration has not consistently kept pace with this expansion globally, and the evidence describing this gap is uneven across regions [3].

A 2024 systematic review found a marked lack of published literature describing vaccination administration training for African pharmacists and student pharmacists, with the limited available evidence concentrated in only a small number of countries [4]. A broader scoping review of pharmacy education in sub-Saharan Africa similarly found the literature concentrated in a few countries, with South Africa and Nigeria contributing the largest numbers of records [5]. In the Middle East, a 2024 cross-sectional study found considerable variation in vaccination-related curricular content and delivery across pharmacy programmes [6].

Where training is absent, the consequences can be significant. In Nigeria, the lack of standardised vaccination administration training has been identified as a barrier limiting pharmacists’ contribution to national immunisation efforts, despite broad professional interest in expanding their role [7]. Similar patterns have been reported elsewhere in Africa. In Lagos and Oyo States, Nigeria, vaccination training was the only significant predictor of whether community pharmacists provided vaccination services, yet only 40.1% had undergone such training [8]. In national surveys of community pharmacists in Ethiopia and Uganda, lack of authorisation was the most frequently reported barrier to pharmacy-based vaccination (94.1% and 95.2%, respectively), alongside training-related barriers [9,10].

Elsewhere, curriculum development efforts have emerged independently in many countries. In Australia, a vaccination training programme combining online learning, simulation, and augmented reality improved student knowledge, confidence, and clinical assessment performance [11]. In Germany, where pharmacists were only recently authorised to administer influenza and COVID-19 vaccines, a high-fidelity simulation vaccination course was developed and evaluated for undergraduates [12] and subsequently found to be adopted inconsistently as a compulsory component across universities [13]. In Belgium, a comparable simulation-based course followed the post-pandemic authorisation of community pharmacists to vaccinate [14], and in Canada, a national webinar-based intervention improved student knowledge, confidence, and preparedness across seven pharmacy schools [15].

The United States provides an example of a long-standing pharmacist vaccination role. Involvement has evolved considerably since early roles limited to vaccine storage and distribution. The first pharmacist immunisation training programme was introduced in 1994, and by 2009 all 50 states permitted pharmacists to administer vaccines [16]. The Accreditation Council for Pharmacy Education (ACPE)’s Standards 2025 require that graduates be prepared to evaluate immunisation needs, administer vaccines safely, and provide evidence-based vaccine counselling [17]. The ACPE standards also inform accreditation processes in other countries. However, even in the US, curricular preparation for paediatric vaccination specifically has lagged. A national survey found that 72% of US schools and colleges of pharmacy did not offer paediatric-focused immunisation training, despite pharmacists’ expanded scope to vaccinate paediatric patients under the Public Readiness and Emergency Preparedness Act [18]. When offered, paediatric training most commonly addressed administration strategies and injection technique, and the most frequently reported barriers were limited curriculum space, cost, facilitator comfort, and instructor workload [18].

Where training programmes have been developed and evaluated, evidence suggests they are effective. Structured, competency-based training, including active-learning laboratory formats and brief just-in-time modules, has prepared pharmacy students to administer both adult and paediatric vaccines [19,20]. This has translated into real-world contributions: student pharmacists have run supervised vaccination clinics with patient satisfaction comparable to non-pharmacy settings [21], and pharmacy interns played a documented role in large-scale COVID-19 immunisation efforts, increasing both vaccine access and their own comfort with administration [22]. Professional organisations, including FIP, have advocated for expanding pharmacist and student pharmacist authority to vaccinate children, and curricular integration of paediatric training has been shown to improve student knowledge and confidence [1,23,24].

These findings suggest a fragmented evidence base. Individual institutions and countries have each independently developed, evaluated, and reported on their own vaccination training programmes, almost always as single-site or single-region studies, while formal accreditation expectations have grown in parallel. A global, cross-regional comparison across pharmacy degree levels is needed that is capable of determining whether the gaps observed reflect a worldwide pattern, or are offset by more developed models elsewhere [3,4,6,25].

The purpose of this study was to gather and evaluate the content, delivery format, assessment model, and certification practices for vaccination administration training across Bachelor’s (BPharm), Master’s (MPharm), and Doctoral (PharmD) pharmacy programmes globally; to assess pharmacy educators’ perceptions of the adequacy of current training; and to identify institutional barriers limiting access to vaccination education.

## 2. Materials and Methods

### 2.1. Study Design and Companion Manuscript

This cross-sectional, self-administered survey study describes the content, delivery format, assessment, and certification practices for vaccination administration training across BPharm, MPharm, and PharmD degree programmes globally, and pharmacy educators’ perceptions of the adequacy of current training. Data reported here were collected as part of a broader survey of pharmacy educators addressing both curriculum content and regulatory/practice conditions. Findings related to pharmacist and pharmacy student scope of practice and perceived benefits of pharmacists administering vaccinations are reported in a companion. The two manuscripts report distinct, non-overlapping survey items and were designed to be read independently.

### 2.2. Instrument Development

Survey items were developed based on findings from a prior systematic review of immunisation administration training for African pharmacists and student pharmacists [4], which identified specific curricular content areas, structural features, and gaps warranting further investigation at a global scale. The draft instrument was piloted with peers in the pharmacy vaccination education field from six different countries (South Africa, Australia, United States, United Kingdom, Nigeria, and the United Arab Emirates) representing five continents; piloting informed refinement of question wording and confirmed an estimated completion time of approximately six minutes.

The final survey instrument comprised nine blocks totalling approximately 47 items, administered via the Qualtrics Survey Platform (Qualtrics XM) with built-in skip logic to route respondents past inapplicable items. The blocks addressed the following: demographic items; pharmacist scope of practice; pharmacy student scope of practice; perceived benefits of pharmacist vaccination; vaccination training content and structure for BPharm, MPharm, and PharmD degree programmes, each as a separate block; vaccination training for practising pharmacists; educator perceptions of training adequacy and institutional barriers. This manuscript reports findings from a subset of the full instrument, specifically demographic items, vaccination training content and structure for BPharm, MPharm, and PharmD degree programmes, vaccination training for practising pharmacists, and educator perceptions of training adequacy and institutional barriers. These items are presented in full in Table 1. Findings from the remaining blocks are reported separately.

### 2.3. Sampling Frame and Recruitment

The International Pharmaceutical Federation (FIP) maintains the World List of Pharmacy Schools as a publicly available registry of pharmacy education institutions worldwide [26]. A sampling method was constructed using FIP’s publicly available World List of Pharmacy Schools, which was used to identify pharmacy programmes in each country. At the time of access (January 2026), this list included pharmacy schools in 146 countries.

For countries with 20 or fewer listed schools, contact identification was attempted for every listed institution. For countries with more than 20 listed schools (e.g., Brazil, the United States), a purposive subset of the top 20 schools was identified using Microsoft Copilot (Microsoft Corporation, Redmond, WA, USA, version M365), an AI-assisted search tool, to prioritise institutions based on national rankings for contact identification. This approach was adopted to achieve a sample that was representative of pharmacy education within high-volume countries, rather than attempting fully comprehensive coverage of every institution in those countries, which was not feasible within the scope of this study.

For each targeted institution, Microsoft Copilot was used to assist in identifying potential participants. A tiered approach was used to prioritise contacts most likely to respond. Copilot was first asked to identify the email address of a pharmacy college or school leader (e.g., dean, department chair, department head, or director) known to be involved in pharmacy education or practice. Where a leadership contact could not be identified, Copilot was instead asked to identify an admissions email address, on the assumption that such addresses are more likely to be monitored regularly than a general institutional contact address. General institutional contact email addresses were deliberately avoided, based on the researcher’s prior experience that such addresses are often unmonitored or disregarded. The aim throughout was to direct recruitment emails to a named individual or a role-specific address likely to receive regular attention, rather than to a generic institutional inbox. In some cases, Copilot was able to identify a relevant institutional website but not a specific contact email address; in these instances, contact information was located manually. Where contact information could not be identified through this process (which occurred for institutions in some countries), an alternative approach was used: authors of published literature on pharmacy education from the country in question were identified and contacted as potential participants. This supplementary approach helped to reduce gaps in country coverage that would otherwise have resulted from limited institutional contact information. Using this combined approach, a total of 823 recruitment emails representing all 146 countries in the FIP list were distributed via the Qualtrics Survey Platform, constituting the potential respondent pool for this study.

Recruitment emails were sent directly to identified contacts, briefly describing the study’s purpose and inviting completion of a survey estimated to take approximately six minutes. Recipients who were not familiar with the vaccination education provided at their institution were asked to forward the survey to a colleague better positioned to respond. This approach was intended to ensure that responses reflected the perspective of an individual with direct knowledge of their institution’s curriculum, though it also means that the eventual respondent at a given institution may not be the originally identified contact. Recipients were also provided with an option to opt out of future emails, which is an option offered by Qualtrics that is automatically imbedded in the recruitment email.

Recruitment was conducted in sequential batches organised by continent between January and April 2026. Each batch remained open for three weeks, with a reminder email sent to non-respondents one week after initial distribution. The reminder email restated the purpose of the survey, the approximate six-minute completion time, and the option to forward the survey to a colleague and thanked those who had already responded.

### 2.4. Data Collection

The survey was administered electronically using the Qualtrics Survey Platform (Qualtrics, Provo, UT, USA). Recruitment emails directed recipients to a survey link, which opened to a consent page presenting the study’s purpose, the primary investigator’s contact information, and the researchers’ institutional affiliation (Nelson Mandela University). The consent page informed potential participants that participation was voluntary, that they could withdraw at any time, that responses would remain anonymous and confidential, that non-mandatory items could be skipped, and that only data from completed questionnaires would be analysed. Potential participants were asked to indicate whether they consented to participate (“Yes, I consent to participate in the survey” or “No, I do not consent to participate in the survey”); only those who selected “Yes” proceeded to the survey itself, while those who selected “No” were directed to a page thanking them for their time.

### 2.5. Quantitative Data Analysis

Survey responses were exported from Qualtrics to Microsoft Excel (version 2608) and analysed descriptively (frequencies and percentages) for categorical items. Since some items were only relevant based on prior responses, the number of respondents (*n*) varies between survey items. The relevant *n* is therefore reported for each result.

Since some response categories had few respondents, inferential statistics appropriate for small cell frequencies were used. Cochran’s Q test would have been the appropriate test to compare the three degree levels, however this was not feasible due to the small sample sizes for Doctoral degrees. Instead, the MPharm and PharmD degree programmes were combined into a single MPharm and PharmD category, called hereafter ‘postgraduate’, so the statistical significance of results could be established using McNemar’s exact test. To obtain statistics regarding the practical significance (effect size) of results, McNemar’s Odds Ratio (OR) was used. Results of inferential tests were considered significant only where they were both statistically significant (*p* < 0.05) and practically significant (McNemar OR ≤ 0.90 or McNemar OR ≥ 1.10).

Paired comparisons within respondents (e.g., adequacy ratings for adult versus paediatric vaccination education, both rated by the same respondent on the same 5-point Likert scale) were analysed using the Wilcoxon signed-rank test, with effect size calculated as *r* (Z/√N) and interpreted using conventional small/moderate/large thresholds [27,28]. A *p*-value less than 0.05 was considered statistically significant.

### 2.6. Ethical Approval

This study was approved by the Research Ethics Committee (Human) at Nelson Mandela University (reference number H23-HEA-PHA-007). All participants were presented with the informed consent information described in Section 2.4 prior to distributing the survey.

## 3. Results

From the 823 recruitment emails, 108 responses (13.1%) were received. After excluding responses from participants who chose non-consent or submitted blank responses, 81 usable responses (9.8%) were received.

### 3.1. Demographic Profile of Respondents

Respondents (*n* = 81) represented pharmacy educators from 35 countries (24%) across all six FIP regions, with the largest representation from the European Region (12 countries) and African Region (10 countries) as shown in Table 2. Most respondents had worked at their current institution for more than 10 years (53.7%), and just over half identified as female (53.1%).

### 3.2. Vaccination Training by Pharmacy Degree Level

Responses organized by degree programme are displayed in Table 3. MPharm and PharmD degree programme responses were combined into a single postgraduate category and compared with Bachelor’s using McNemar’s exact test for each variable. Training status (taught vs. not taught) did not differ significantly between Bachelor’s (41.9% taught) and postgraduate (32.4% taught) levels (McNemar χ^2^(1, *n* = 74) = 1.33, *p* = 0.248, OR = 0.59); although the odds ratio reflected a moderate effect, the result did not reach statistical significance. Neither paediatric technique inclusion (McNemar exact *p* = 1.000, *n* = 10) nor travel vaccine content inclusion (McNemar exact *p* = 1.000, *n* = 11) differed significantly between levels; odds ratios could not be calculated for either comparison because too few respondents gave discordant answers between levels. Post-training vaccination authority (permitted vs. not permitted) also did not differ significantly between levels (McNemar test not computable, *n* = 13; all matched respondents gave the same answer at both levels).

### 3.3. Who Delivers Vaccination Training

In-house faculty delivered most of the training at every degree level (81.0–87.5%) as shown in Table 4. Reliance on local practising pharmacists facilitating the training rose from 24.2% at Bachelor’s level to 50.0% at Doctoral level.

Among the subset of institutions reporting instructor information at all three degree levels (*n* = 13), Bachelor’s and postgraduate responses were identical for every instructor category, so McNemar’s test could not be computed for any of the six comparisons (no respondents gave discordant answers between levels). This suggests that institutions using a given instructor type at the Bachelor’s level tended to use the same instructor type for postgraduate training, and vice versa.

### 3.4. Assessment/Pass Criteria

A required written knowledge assessment used to evaluate student learning was similar across all three levels (75.0–77.4%) among institutions offering vaccination training for each degree programme. The proportion of programmes requiring students to demonstrate technique by injecting another person, rather than a device alone, declined from 48.4% at Bachelor’s level to 25.0–30.0% at postgraduate level, as shown in Table 5.

None of the four assessment types differed significantly between Bachelor’s and postgraduate levels among institutions reporting on all three degree levels (*n* = 13; McNemar exact *p* = 1.000 for all four comparisons). Odds ratios could not be calculated for any comparison because too few respondents gave discordant answers between levels (fewer than five).

### 3.5. Certification

Roughly half of students at every degree level completed training without receiving any formal certificate, and certification from a regulatory body was uncommon across all three levels, as shown in Table 6. Certificate source did not differ significantly between Bachelor’s and postgraduate levels for any of the four categories among institutions reporting on all three degree levels (*n* = 13; McNemar exact *p* = 1.000 for all comparisons that could be computed). Odds ratios could not be calculated because of too few discordant respondents, and the regulatory-body certificate comparison could not be tested at all, as no respondent reported receiving this certificate at either level.

### 3.6. Vaccination Training for Practising Pharmacists

Only nine (12.0%) of the responding institutions offered any structured immunisation training short course for practising pharmacists. Among the eight institutions reporting assessment details, all required a written or online examination. Practical assessment, including injection on a device, manikin, or a person, was less common. Only half included paediatric administration technique, and one-third reported that no certificate was received. Results for survey items related to short course training for practising pharmacists are shown in Table 7. 

### 3.7. Educator Perceptions of Training Adequacy and Expansion Plans

Educators rated their institution’s vaccination education as less adequate for paediatric patients than for adults and were more optimistic about future expansion of training for practising pharmacists than for students, as shown in Table 8. Educators rated their institution’s education as significantly less adequate for preparing students to vaccinate paediatric patients than adult patients (Wilcoxon signed-rank *W* = 5.5, *n* = 71, *p* < 0.001, r = 0.52—a large effect), despite both ratings coming from the same respondents evaluating the same institution. Expansion plans followed a similar pattern: respondents were somewhat more likely to report plans to expand vaccination education for practising pharmacists (50.7% agree/strongly agree, *n* = 36) than for pharmacy students (47.9%, *n* = 34), though this difference was not formally tested.

### 3.8. Barriers to Vaccination Education

Financial cost and competition for curriculum time were the most frequently cited barriers to vaccination education, while lack of access to supplies was not reported as a barrier by any respondent. Financial barriers, competing curriculum demands, and limited access to training were each reported by more than a quarter of respondents, while institutional approval was cited as a barrier as often as access to training itself. Student fear or anxiety was a comparatively minor barrier, and no respondent reported a lack of access to supplies. Results for survey items related to barriers are shown in Table 9.

## 4. Discussion

This study surveyed international pharmacy educators and was, to our knowledge, the first to compare vaccination administration training across BPharm, MPharm, and PharmD degree programmes in multiple countries, surveyed pharmacy educators from 35 countries (*n* = 81). Vaccination administration was not taught at most institutions at any degree level (56.4% BPharm, 71.2% MPharm, 89.0% PharmD). The most robust finding was a paediatric-specific gap: among institutions offering training, fewer than a third included paediatric administration technique at any degree level (28.6% BPharm, 14.3% MPharm, 20.0% PharmD). Respondent educators rated their institution’s preparation as significantly less adequate for paediatric than adult vaccination (*r* = 0.52). Roughly half of students at every degree level completed training without a formal certificate. Financial cost (41.4%) and limited curriculum time (37.1%) were the most frequently reported barriers. These findings are consistent with gaps previously reported in the Middle East and Africa [4,6].

### 4.1. Vaccination Training Is Absent at Most Respondent Institutions and Appears to Be Less Common at Higher Degree Levels

Vaccination administration was not taught at most responding institutions, and the proportion not teaching it rose with degree level (Table 3). Among the 74 respondents who provided data for both BPharm and postgraduate (MPharm and PharmD programme levels, training was taught at 41.9% (31/74) of Bachelor’s programmes and 32.4% (24/74) of postgraduate programmes. This difference was not statistically significant (McNemar χ^2^(1, *n* = 74) = 1.33, *p* = 0.248, OR = 0.59), and the figures differ from the Bachelor’s column of Table 3 (*n* = 78) because the paired analysis is restricted to respondents with data at both levels. The gradient across degree levels in Table 3 should therefore be read as descriptive rather than confirmed. The absence of a statistically confirmed differences warrants further study in a larger, degree-level-balanced sample.

Results may also be a reflection of legal authority rather than curricular choice. Where pharmacists or students are not permitted to vaccinate, our finding of “not taught” may be a rational response rather than a gap. Authority does not appear to explain the pattern completely, however. Among respondents who answered the post-training authority item, 50.0% at BPharm level, 40.0% at MPharm level, and 25.0% at PharmD level reported that graduates were still not permitted to vaccinate after training, suggesting that some programmes teach administration ahead of, or without, corresponding authority. The cross-sectional design and small number of respondents per country do not allow us to separate these explanations. Separate studies of practising pharmacists in Africa point in the same direction. Lack of authorisation was the most frequently reported barrier to pharmacy-based vaccination in national surveys in Ethiopia (94.1%) and Uganda (95.2%) [9,10], and stakeholders in Nigeria reported that no law either restricted or authorised community pharmacists to vaccinate, describing COVID-19 vaccination authority as a one-off arrangement [29]. Together, these suggest that authority and training are interdependent rather than separable barriers.

Numerically, the most clinically advanced degree levels were, if anything, less likely to include vaccination training. Among institutions that did offer training, the proportion of programmes requiring students to demonstrate technique on an actual person, rather than a device alone, declined from 48.4% at BPharm level to 25.0 at MPharm level and 30.0% PharmD level, suggesting that the more clinically realistic form of assessment becomes rarer as training progresses. Larger, degree-level-balanced samples would be needed to determine whether postgraduate training is truly less common and, if so, whether this reflects curricular oversight, assumed competency from undergraduate training, or structural barriers specific to postgraduate curricula.

Two contrasting examples illustrate the disconnect between authority and training. In Germany, where pharmacists were only recently authorised to administer influenza and COVID-19 vaccines, a simulation-based course was developed for undergraduates [12] but was subsequently found to be adopted inconsistently as a compulsory component across universities [13]. In Nigeria, training was the only significant predictor of whether community pharmacists provided vaccination services, yet only 40.1% had undergone vaccination training [8]. Health systems in these two countries differ considerably, but both show the same gap between authority and training preparation evident in our data. This suggests the gaps are not specific to one region, although 81 self-selected respondents cannot establish how common they are within any country. Closing them is unlikely to be achieved through curriculum reform alone. Expanding a prepared vaccination workforce typically requires coordinated action across governments, regulatory bodies, educational institutions, and public health agencies [30], and training without integration into national immunisation planning may have limited impact on workforce readiness [31].

### 4.2. Paediatric Vaccination Training: A Persistent Gap Even in Established Systems

Paediatric-focused training in particular lags behind the expansion of pharmacist vaccination authority, and this pattern is not confined to countries with newer or less-resourced vaccination systems. Educators rated their institution’s education as significantly less adequate for preparing students to vaccinate paediatric patients than adult patients (Wilcoxon signed-rank *W* = 5.5, *p* < 0.001, *r* = 0.52—a large effect), a striking gap given that both ratings came from the same respondents evaluating the same institution. This mirrors findings from a national survey of US pharmacy schools [18], where 72% did not offer paediatric-focused immunisation training despite pharmacists’ expanded scope to administer paediatric vaccines under the Public Readiness and Emergency Preparedness Act. Where offered, paediatric vaccination training in the US most commonly addressed administration strategies and correct injection technique, and the most frequently reported barriers were limited curriculum space, cost, facilitator comfort, and instructor workload [18]. That the same barriers recur in a country with one of the most established pharmacist vaccination authorities suggests they are structural rather than a symptom of newer or less-resourced systems, and that expanded scope-of-practice legislation alone is unlikely to close the gap without investment in curricular capacity. Inconsistent preparation of the pharmacy workforce for paediatric vaccination has implications for vaccine-preventable disease burden that extend well beyond individual training programmes.

Systematic reviews of pharmacist involvement in immunisation have consistently found that the strongest evidence comes from high-income countries and adult vaccination services, particularly influenza [25,32]. The present findings extend this observation from the delivery side to the training side: even where paediatric authority exists, the pipeline preparing pharmacists and students specifically for paediatric administration remains comparatively underdeveloped. Preceptor feedback on student pharmacists suggests that general confidence and child-comforting technique, rather than injection technique, are the areas most in need of improvement [33]. Since the paediatric shortfall is the most robust and actionable finding of this study, it suggests that pharmacy education would benefit from a standardised minimum competency benchmark for paediatric vaccination training that accreditation bodies could incorporate directly into curricular standards, rather than leaving paediatric content as an optional addition to adult-focused training.

### 4.3. Barriers, Certification, and Pathways to Training Expansion

The barriers reported by educators help explain why paediatric-specific training in particular remains underdeveloped. Results suggest that institutional and structural constraints are more problematic than learner readiness. Student fear or anxiety was reported by only 15.7% of respondents. Costs, lack of curricular time, access to training, and a lack of leadership approval were reported more frequently by respondents. These results may indicate that programmes lack access to training because leadership has not prioritised or approved it, but also that leadership may withhold approval because accessible, affordable training is unavailable. About half of respondents anticipated expanding vaccination education for practising pharmacists and pharmacy students, and expansion is more likely to succeed if these barriers are addressed directly.

Since roughly half of students at every degree level completed training without a formal certificate, institutions and professional bodies should consider whether certification should be an explicit, required outcome of training rather than left to individual programme discretion. Pathways of this kind exist. In Nigeria, stakeholders reported that vaccination delivery has been added to the regulator’s mandatory continuing professional development modules, and more than 700 community pharmacists were trained through related programmes [29].

### 4.4. Strengths and Limitations

This study has several strengths. It draws on pharmacy educators from 35 countries, allowing training practices to be compared across multiple regulatory and educational jurisdictions rather than within a single country or region. To our knowledge, no previous study has done this across BPharm, MPharm, and PharmD programmes. Second, the survey examined several components of training within a single instrument, including content, delivery format, assessment, certification, barriers, and perceived adequacy. It also distinguished paediatric from adult vaccination, which is what allowed the paediatric-specific gap to be identified. Third, degree-level comparisons used matched responses from the same institutions and a test suited to small, paired samples, so differences between levels were not confounded by differences between institutions. Finally, respondents were educators with direct knowledge of their own programmes’ curricula.

This study also has several limitations. Of 823 recruitment emails distributed, 108 responses were recorded. After excluding one respondent who did not consent to participate, 25 records containing no substantive responses beyond country and institution, and one test record, 81 usable responses remained, corresponding to a completed-response rate of 9.8%. This is consistent with response rates typically observed in cold-outreach international survey research, but it means the findings reported here reflect a self-selected subset of contacted institutions rather than a representative sample of global pharmacy education generally.

Country-level coverage was also uneven. With 81 respondents across 35 countries, most countries were represented by only one or two respondents, meaning findings attributed to a given country reflect the experience of a single institution rather than that country’s pharmacy education landscape as a whole. Similarly, for countries with more than 20 listed pharmacy schools, recruitment targeted a purposive subset of 20 institutions rather than the full population of schools, which may not capture the full range of training practices within those countries. The degree-level categories may correspond to different programme structures across countries, which may partly explain the low prevalence reported at postgraduate levels.

All data were self-reported by pharmacy educators describing their own institution’s curriculum, without independent verification against course syllabi, accreditation records, or other institutional documentation; responses may therefore be subject to recall error or social desirability bias. Training status was not adjusted for national regulatory context. The survey instrument was administered in English only, which may have limited participation from pharmacy educators at institutions where English is not the primary language of instruction or professional communication, potentially underrepresenting non-English-speaking regions.

Since several countries were represented by only one or two respondents and country and institution were recorded at the time of response, respondents could in principle be identifiable in countries with few schools. To limit this risk, identifying details were separated from survey responses before analysis and the dataset is not publicly available, in accordance with ethics committee restrictions. Nonetheless, the small number of respondents per country may have made some educators less willing to respond candidly, particularly about barriers or leadership approval, which may have introduced social desirability bias in either direction.

Finally, several inferential comparisons were based on small, matched subsamples (*n* = 10–13), reflecting the number of respondents who provided complete data across all three degree levels for a given item. While McNemar’s exact test and odds ratio are appropriate for this data structure, these subsample sizes limit statistical power, and several comparisons could not be tested at all due to the insufficient variation in responses. These results should be interpreted as preliminary rather than conclusive, and larger, degree-level-balanced samples would be needed to confirm whether the patterns observed here, particularly the numerical gap between BPharm and postgraduate training, reflect genuine differences.

### 4.5. Implications and Next Steps

These findings point to several concrete next steps for pharmacy education and accreditation bodies. First, the absence of a statistically confirmed difference between BPharm and postgraduate training should not be read as reassurance: the consistent numerical pattern across multiple independent variables (training status, assessment rigour, certification) warrants replication in a larger, degree-level-balanced sample specifically powered to detect these differences, rather than being set aside as a null result. Next, the paediatric-specific training gap identified here is the more robust and actionable finding, and would benefit from a standardised, minimum competency benchmark for paediatric vaccination training that accreditation bodies could incorporate directly into curricular standards, rather than leaving paediatric content as an optional addition to adult-focused training. Third, given that roughly half of trained students at every degree level received no formal certificate, institutions and professional bodies should consider whether certification should be an explicit, required outcome of training rather than left to individual programme discretion. Lastly, future work should examine why postgraduate programmes in particular show numerically lower training prevalence than Bachelor’s-level programmes, since the present study’s cross-sectional design cannot distinguish between curricular oversight, assumed prior competency from undergraduate training, or structural barriers specific to postgraduate curricula.

## 5. Conclusions

This global survey of pharmacy educators across 35 countries found that vaccination administration training remains inconsistent across BPharm, MPharm, and PharmD degree programmes. The most robust finding was a paediatric-specific training gap: fewer than a third of institutions offering training included paediatric administration technique at any degree level, and educators consistently rated their institution’s preparation for paediatric vaccination as less adequate than for adult vaccination. This gap recurred even in a country with among the most established pharmacist vaccination authority in the world, suggesting a structural feature of curricula rather than a symptom of newer or less-resourced systems. Certification was similarly inconsistent, with roughly half of students at every degree level completing training without receiving any formal certificate.

Financial cost and limited curriculum time were the most commonly reported obstacles to training, more so than student readiness or institutional approval alone, suggesting that resource investment will be necessary to close these gaps. Additionally, using a standardised, minimum competency benchmark for paediatric vaccination training incorporated into accreditation standards, alongside clearer certification requirements at the institutional level, could also be beneficial. Larger, degree-level-balanced samples would help confirm whether the numerical gap between Bachelor’s and postgraduate training reflects a pattern worth addressing directly in curriculum design.

## Figures and Tables

**Table 1:** List of survey items and response structure, organized by block.

Survey Block	Survey Items	Response Format and Options
Demographic items	1. In which country is the school or institution where you are employed located? If you teach/work at more than one institution, then please identify the country in which you teach/work most frequently.	Open text
	2. What is the name of the institutions where you are employed? As a reminder, individual responses and names of institutions will not be shared.	Open text
	3. How many years have you been working at your current institution?	4-category (less than 1 year, 1 to 5 years, 6 to 10 years, more than 10 years)
	4. Which gender do you most identify with?	4-category (male, female, non-binary/third gender, prefer not to say)
Vaccination training content and structure for Bachelor’s, Master’s, and Doctoral degree programmes [4]	Is vaccination training taught as part of the [BPharm, MPharm, and PharmD] degree programme at your institution?	Required/Optional/No (skip if No)
	Vaccination training for [BPharm, MPharm, and PharmD] students at my institution is taught by:	Select all that apply (Faculty/professors/staff who are employed by my institution; Faculty/professors/staff from another institution; Representatives of a regional and/or national organization; Local practicing pharmacists; Representatives of a regulatory board/organization; Members of another health discipline (nurses, medical doctors, physicians, etc); None of these)
	Does the vaccination training for [BPharm, MPharm, and PharmD] degree students include paediatric administration technique?	Yes/No/Not sure
	Does the vaccination training for [BPharm, MPharm, and PharmD] degree students include information about travel vaccines?	Yes/No/Not sure
	In order to complete/pass vaccination training, [BPharm, MPharm, and PharmD] degree students must (select all that apply):	Select all that apply (Demonstrate knowledge using a written/online examination; Demonstrate appropriate injection technique by injecting a sponge, manikin, or other practice device; Demonstrate appropriate injection technique by injecting another person; Complete a specified number of vaccinations under the supervision of a qualified vaccinator)
	After completing vaccination training, do [BPharm, MPharm, and PharmD] degree students receive a certificate to be able to vaccinate?	4-category (Yes, they receive certificate from my institution; Yes, they receive certificate from a pharmacy organisation; Yes, they receive certificate from a regulatory body; No)
	After completing vaccination training, [BPharm, MPharm, and PharmD] degree students	4-category (May begin administering vaccinations immediately under the supervision of a pharmacist; May begin administering vaccinations immediately without supervision; May begin administering vaccinations after graduation from the pharmacy programme; Are still not allowed to vaccinate)
Vaccination training for practising pharmacists [4]	Does your institution offer an immunisation short course or supplementary training for practicing pharmacists?	Yes/No (skip if No)
	Vaccination training for practicing pharmacists your institution is taught by (please select all that apply):	Select all that apply (Faculty/professors/staff who are employed by my institution; Faculty/professors/staff from another institution; Representatives of a regional and/or national organization; Local practicing pharmacists; Representatives of a regulatory board/organization; Members of another health discipline (nurses, medical doctors, physicians, etc); None of these)
	Does the vaccination training for practicing pharmacists at your institution include paediatric administration technique?	Yes/No/Not sure
	Does the vaccination training for practicing pharmacists at your institution include information about travel vaccines?	Yes/No/Not sure
	In order to complete/pass vaccination training, practicing pharmacists must (select all that apply):	Select all that apply (Demonstrate knowledge using a written/online examination; Demonstrate appropriate injection technique by injecting a sponge, manikin, or other practice device; Demonstrate appropriate injection technique by injecting another person; Complete a specified number of vaccinations under the supervision of a qualified vaccinator)
	After completing vaccination training, do practicing pharmacists receive a certificate?	4-category (Yes, they receive certificate from my institution; Yes, they receive certificate from a pharmacy organisation; Yes, they receive certificate from a regulatory body; No)
Educator perceptions of training adequacy and institutional barriers [4]	Rate your level of agreement with the following statement: The education for students enrolled at the institution where I work is appropriate for preparing them to administer vaccinations to adults.	5-point Likert Scale (1—Strongly Disagree; 2—Disagree; 3—Neutral; 4—Agree; 5—Strongly Agree)
	Rate your level of agreement with the following statement: The education for students enrolled at the institution where I work is appropriate for preparing them to administer vaccinations to paediatric patients.	5-point Likert
	Rate your level of agreement with the following statement: The country where I teach/work has plans to improve/expand vaccination education initiatives for pharmacy students.	5-point Likert
	Rate your level of agreement with the following statement: The country where I teach/work has plans to improve/expand vaccination education initiatives for practicing pharmacists. (4)	5-point Likert
	Please select from the list below any barriers that exist at the institution where you teach/work that limit access to vaccination education for student pharmacists.	Select all that apply (Lack of time in curriculum; Lack of access to supplies; Lack of access to training; Financial barriers such as cost of trainer or supplies; Fear or anxiety of students to learn these skills; Lack of approval from programme leadership; Other-please describe)

Abbreviations used: BPharm: Bachelor of Pharmacy; MPharm: Master of Pharmacy; PharmD: Doctor of Pharmacy.

**Table 2:** Demographic characteristics of survey respondents.

Characteristic	*n*	%
Years at institution (*n* = 67)		
Less than 1 year	1	1.5
1–5 years	13	19.4
6–10 years	17	25.4
More than 10 years	36	53.7
Gender (*n* = 64)		
Female	34	53.1
Male	30	46.9
FIP region (*n* = 81)		
Africa: Algeria (1), Botswana (1), Eswatini (1), Ghana (5), Kenya (1), Nigeria (2), Senegal (1), Sierra Leone (1), South Africa (1), Zambia (1).	15	18.5
Americas: Brazil (1), Canada (7), United States (2).	10	12.3
Eastern Mediterranean: Jordan (2), Lebanon (2), United Arab Emirates (2), Yemen (2).	8	9.9
Europe: Bosnia and Herzegovina (1), Cyprus (2), Denmark (2), Georgia (2), Ireland (2), Kosovo (1), Malta (1), Netherlands (2), Norway (1), Portugal (4), Türkiye (4), United Kingdom (3)	25	30.9
South-East Asia: India (4), Indonesia (2).	6	7.4
Western Pacific: Australia (10), Cambodia (2), Malaysia (4), New Zealand (1).	17	21.0

**Table 3:** Vaccination training characteristics by degree level.

Item	BPharm (*n*, %)	MPharm (*n*, %)	PharmD (*n*, %)
Training status	(*n* = 78)	(*n* = 73)	(*n* = 73)
Not taught	44 (56.4)	52 (71.2)	65 (89.0)
Taught as a required component	24 (30.8)	13 (17.8)	3 (4.1)
Taught as an optional component	10 (12.8)	8 (11.0)	5 (6.8)
Training includes paediatric technique *	(*n* = 28)	(*n* = 14)	(*n* = 5)
Taught	8 (28.6)	2 (14.3)	1 (20.0)
Not taught	20 (71.4)	12 (85.7)	4 (80.0)
Training includes travel vaccine content *	(*n* = 28)	(*n* = 18)	(*n* = 6)
Taught	19 (67.9)	11 (61.1)	5 (83.3)
Not taught	9 (32.1)	7 (38.9)	1 (16.7)
Post-training, vaccination authority is	(*n* = 32)	(*n* = 20)	(*n* = 8)
Still not permitted	16 (50.0)	8 (40.0)	2 (25.0)
Permitted after graduation	6 (18.8)	7 (35.0)	2 (25.0)
Permitted immediately, supervised	9 (28.1)	4 (20.0)	3 (37.5)
Permitted immediately, unsupervised	1 (3.1)	1 (5.0)	1 (12.5)

* Among institutions offering training at that degree level.

**Table 4:** Instructors delivering vaccination training, by degree level (select all that apply).

Taught By	BPharm (*n*, %) (*n* = 33)	MPharm (*n*, %) (*n* = 21)	PharmD (*n*, %) (*n* = 8)
Faculty/staff employed by my institution	28 (84.8)	17 (81.0)	7 (87.5)
Local practising pharmacists	8 (24.2)	7 (33.3)	4 (50.0)
Members of another health discipline	8 (24.2)	10 (47.6)	2 (25.0)
Faculty/staff from another institution	4 (12.1)	1 (4.8)	1 (12.5)
Regional/national organisation representatives	4 (12.1)	1 (4.8)	1 (12.5)
Regulatory board representatives	2 (6.1)	1 (4.8)	0 (0.0)
None of these	1 (3.0)	1 (4.8)	0 (0.0)

**Table 5:** Assessment/pass criteria for vaccination training, by degree level (select all that apply).

To Pass, Students Must…	BPharm (*n*, %) (*n* = 31)	MPharm (*n*, %) (*n* = 20)	PharmD (*n*, %) (*n* = 8)
Demonstrate knowledge using a written/online examination	24 (77.4)	15 (75.0)	6 (75.0)
Demonstrate appropriate injection technique by injecting a sponge, manikin, or other practice device	22 (71.0)	14 (70.0)	5 (62.5)
Demonstrate appropriate injection technique by injecting another person	15 (48.4)	6 (30.0)	2 (25.0)
Complete a specified number of vaccinations under the supervision of a qualified vaccinator	9 (29.0)	5 (25.0)	2 (25.0)

**Table 6:** Certificate received after training, by degree level.

Certificate Source	BPharm (*n*, %) (*n* = 32)	MPharm (*n*, %) (*n* = 21)	PharmD (*n*, %) (*n* = 8)
No certificate	17 (53.1)	11 (52.4)	3 (37.5)
From my institution	9 (28.1)	8 (38.1)	2 (25.0)
From a pharmacy organisation	5 (15.6)	3 (14.3)	2 (25.0)
From a regulatory body	4 (12.5)	0 (0.0)	1 (12.5)

**Table 7:** Vaccination training short course for practising pharmacists.

Item	*n*	%
Short course offered (*n* = 75)		
Yes	9	12.0%
No	66	88.0%
Includes paediatric technique * (*n* = 4)		
Yes	2	50.0%
No	2	50.0%
Includes travel vaccine content * (*n* = 5)		
Yes	2	40.0%
No	3	60.0%
Taught by * (*n* = 9, select all that apply)		
Faculty/staff employed by my institution	7	77.8%
Members of another health discipline	2	22.2%
Faculty/staff from another institution	1	11.1%
Regional/national organisation representatives	1	11.1%
Local practising pharmacists	1	11.1%
Regulatory board representatives	1	11.1%
None of these	1	11.1%
To pass, pharmacists must complete a * (*n* = 8, select all that apply)		
Written/online examination	8	100.0%
Injection on device/manikin	4	50.0%
Complete supervised vaccination count	4	50.0%
Injection on a person	2	25.0%
Certificate received * (*n* = 9)		
From my institution	5	55.6%
From a regulatory body	3	33.3%
No certificate	3	33.3%
From a pharmacy organisation	0	0.0%

* Among institutions offering a short course.

**Table 8:** Educator perceptions of training adequacy and expansion plans (*n* = 71).

Survey Item	Disagree or Strongly Disagree		Neutral		Agree or Strongly Agree	
	*n*	%	*n*	%	*n*	%
The education for students enrolled at the institution where I work is appropriate for preparing them to administer vaccinations to adults.	27	38.0%	10	14.1%	34	47.9%
The education for students enrolled at the institution where I work is appropriate for preparing them to administer vaccinations to paediatric patients.	37	52.1%	15	21.1%	19	26.8%
The country where I teach/work has plans to improve/expand vaccination education initiatives for pharmacy students.	17	23.9%	20	28.2%	34	47.9%
The country where I teach/work has plans to improve/expand vaccination education initiatives for practising pharmacists.	13	18.3%	22	31.0%	36	50.7%

**Table 9:** Barriers limiting access to vaccination education (*n* = 70).

Barrier	No		Yes	
	*n*	%	*n*	%
Financial barriers (e.g., cost of trainer or supplies)	41	58.6	29	41.4
Lack of time in curriculum	44	62.9	26	37.1
Lack of access to training	50	71.4	20	28.6
Lack of approval from programme leadership	50	71.4	20	28.6
Fear of anxiety of students to learn these skills	59	84.3	11	15.7
Lack of access to supplies	70	100.0	0	0.0

## Data Availability

The datasets presented in this article are not readily available because of restrictions from the Research Ethics Committee (Human). Requests to access the datasets should be directed to the corresponding author.

## Abbreviations

The following abbreviations are used in this manuscript:

ACPE	Accreditation Council for Pharmacy Education
BPharm	Bachelor of Pharmacy
COVID-19	Coronavirus Disease 2019
FIP	International Pharmaceutical Federation
MPharm	Master of Pharmacy
OR	Odds Ratio
NMU	Nelson Mandela University
PharmD	Doctor of Pharmacy
